# On Phlegmatia Dolens

**Published:** 1831-01-01

**Authors:** 


### XXXIX.

**Case of Phlegmatia Dolens, caused by Inflammation of the Veins of the Lower Extremity, excited by malignant Ulceration of the Cervix Uteri. By William Lawrence, Esq.**

[Med.-Chir. Trans, Vol. XVI.

Although we are very far from agreeing with Mr. Lawrence, that " the following case *completely confirms* the interesting and important observations respecting the nature and causes of phlegmatia dolens, lately communicated to the Society by Robert Lee"—and for this simple reason, that we do not consider the case as one of phlegmatia dolens at all—yet we shall give it in the words of the author.

" Anne Dawson, forty years of age, a married woman, who had borne several children, was received into St. Bartholomew's Hospital under my care, on the 12th of November, 1829. Her complexion was sallow, and the expression of the countenance altogether very unhealthy. She had pain in the loins, frequently shooting towards the hypogastric region, which was tender on pressure; costive bowels; restlessness; and sanious discharge from the vagina. She had not menstruated for several months. For the last six months she had laboured under incontinence of urine; she had perfect use of her legs, and full power over the sphincter ani. There was no tenderness in the region of the spine. Instead of the os tincæ and cervix uteri, a large irregular ulcerated excavation was found at the posterior end of the vagina. Anodynes and the occasional use of castor oil were directed, and afforded some relief.

About the 30th of November, increased uneasiness was experienced in the lower part of the abdomen, with feverish symptoms not of a severe description; the pulse was sharp and frequent, the tongue white, the skin warm, and the countenance slightly flushed. The right lower extremity swelled in its whole extent, with some increase of heat, and pain on motion, which was performed with difficulty. The colour of the limb was not altered; the swollen part of the thigh was tolerably firm, the lower part of the leg, and the foot, pitted on *pressure*. There was pain in the course of the femoral and iliac vessels; and the internal saphena vein could be traced at the upper part of the thigh by a hardened knotty feel. I considered the disease to be essentially the same as the Phlegmasia Dolens, occurring in women recently delivered; there could be no doubt that the large veins of the thigh were inflamed; and the observations I had heard from Dr. Lee led me to conclude that inflammation had been excited in the veins of the uterus by the disease in its cervix, and had extended from them to the iliac and femoral venous trunks. I ordered twelve leeches to the front of the thigh, over the course of the femoral vein; that the bleeding from the orifices should be encouraged by fomentation, and the part afterwards covered with a bread poultice. A saline draught, with a drachm of the sulphate of magnesia, was also directed every six hours. On the next day the swelling of the thigh was less; the anasarcous state of the leg and foot remained. There was less pain, and greater freedom of motion. The feverish symptoms were lessened.

The pain returned on the 25th, when the leeches were repeated with benefit. She gradually improved from this time; the tenderness at the lower part of the abdomen being diminished, and the incontinence of urine less troublesome. The latter symptoms, however, with a discharge from the vagina, at first supposed to be menstrual, returned about the 16th of December, and were relieved by alum injections, with the internal use of dilute sulphuric acid and tincture of opium. On the 18th, violent hæmorrhage from the uterus came on early in the morning, and was speedily fatal. A large quantity of blood had flowed before she became aware of the circumstance, and called for assistance.

*Examination*. When the body was examined, on the second day after death, the fundus of the uterus was found moderately enlarged and firm; the cervix had been destroyed by that kind of phagedenic ulceration, which is usually called cancer of the uterus. The rectum and sigmoid flexure of the colon adhered firmly to the uterus; and, but for this adhesion, the ulceration would have penetrated the cavity of the abdomen. The cellular and adipose substance round the lower part of the uterus, and neighbouring portion of the vagina, were thickened and indurated, particularly on the right side. The hypogastric vein, involved in this diseased mass, was closed, in consequence of previous inflammation of its coats; and the same change had occurred in the internal iliac, the common iliac, the external iliac, the femoral and profunda veins, as well as in the internal saphena, all of which were completely impervious. The affection terminated above at the junction of the common iliac vein with that of the opposite side; the latter vessel and the inferior cava being quite natural. The saphena was closed for a length of about four or five inches, beyond which it was natural. The profunda was cut through near the femoral vein, and the latter was divided as it passes the tendon of the triceps. The disease extended in both these vessels beyond the situations where they had been divided; but its inferior limits were not ascertained. The right spermatic vein was closed in its lower half. The coats of the affected vessels and the surrounding cellular substance were a little thickened, and their cavities were plugged by a closely adherent, and tolerably firm substance of a light brown colour. At some parts the vessels and their contents were of a dark livid hue."

To our obstetric brethren who have opportunities of seeing the phlegmatia dolens of lying-in-women, we recommend a careful perusal of the above case, and if they find that it corresponds in history, symptoms, and termination with the disease under which it is classed, then they may conclude that we are entirely wrong in our estimation of the nature and cause of phlegmatia dolens.

